# Differential Reliance on Autophagy for Protection from HSV Encephalitis between Newborns and Adults

**DOI:** 10.1371/journal.ppat.1004580

**Published:** 2015-01-08

**Authors:** Douglas R. Wilcox, Nitin R. Wadhwani, Richard Longnecker, William J. Muller

**Affiliations:** 1 Department of Pediatrics, Northwestern University Feinberg School of Medicine, Chicago, Illinois, United States of America; 2 Department of Microbiology and Immunology, Northwestern University Feinberg School of Medicine, Chicago, Illinois, United States of America; 3 Department of Pathology, Ann & Robert H. Lurie Children's Hospital of Chicago, Chicago, Illinois, United States of America; University of North Carolina at Chapel Hill, United States of America

## Abstract

Newborns are more susceptible to severe disease from infection than adults, with maturation of immune responses implicated as a major factor. The type I interferon response delays mortality and limits viral replication in adult mice in a model of herpes simplex virus (HSV) encephalitis. We found that intact type I interferon signaling did not control HSV disease in the neonatal brain. However, the multifunctional HSV protein γ34.5 involved in countering type I interferon responses was important for virulence in the brain in both age groups. To investigate this observation further, we studied a specific function of γ34.5 which contributes to HSV pathogenesis in the adult brain, inhibition of the cellular process of autophagy. Surprisingly, we found that the beclin binding domain of γ34.5 responsible for inhibiting autophagy was dispensable for HSV disease in the neonatal brain, as infection of newborns with the deletion mutant decreased time to mortality compared to the rescue virus. Additionally, a functional beclin binding domain in HSV γ34.5 did not effectively inhibit autophagy in the neonate, unlike in the adult. Type I IFN responses promote autophagy in adult, a finding we confirmed in the adult brain after HSV infection; however, in the newborn brain we observed that autophagy was activated through a type I IFN-independent mechanism. Furthermore, autophagy in the wild-type neonatal mouse was associated with increased apoptosis in infected regions of the brain. Observations in the mouse model were consistent with those in a human case of neonatal HSV encephalitis. Our findings reveal age-dependent differences in autophagy for protection from HSV encephalitis, indicating developmental differences in induction and regulation of this innate defense mechanism after HSV infection in the neonatal brain.

## Author Summary

Disease after infection with a pathogen results from an intersection between the infectious agent and the host. Newborns are particularly susceptible to infectious illness compared to adults, and HSV infection commonly results in devastating encephalitis. We studied the interaction of HSV with the type I interferon pathway and found that a specific activity of the viral protein γ34.5, which counters host autophagy to promote encephalitis in adults, was not required to cause disease in newborns. Furthermore, autophagy was not inhibited by HSV in the neonate and was not activated by type I interferon signaling, unlike in the adult. Activated autophagy was associated with increased apoptosis, which may contribute to the increased pathology in newborns. Our findings reveal development-specific differences in the pathogenesis of HSV encephalitis, including a distinct role for autophagy in the neonatal brain.

## Introduction

Disease due to viral infection is a complex consequence of interactions between both viral and host factors. Herpes simplex virus (HSV) infections cause a wide spectrum of outcomes in humans, ranging from asymptomatic acquisition to lethal dissemination and encephalitis [1]. Newborns are particularly susceptible to poor neurologic outcomes of central nervous system (CNS) disease from HSV [2]. Over half of neonatal HSV infections result in disseminated disease or encephalitis, with long-term neurologic morbidity in 2/3 of those who survive encephalitis. In contrast, HSV infection in the adult population is often subclinical [3]. Either serotype of HSV may cause disease in newborns (HSV-1 or HSV-2), but emerging data suggests a rising incidence of HSV-1 genital infection [4], and a parallel predominance of HSV-1 as a cause of newborn disease [5], [6]. The disparate outcomes between HSV-infected neonates and adults suggest an age-dependent difference in susceptibility to disease based on host factors. Multiple layers of immunity are involved in the host response to HSV infection, and differences in immune responses of newborns compared with adults likely contribute to their increased susceptibility [7]. Additionally, multiple host signals important in immunity are targeted by the virus for modulation [8], and it is not clear how HSV may manipulate these responses differently in the newborn.

The HSV γ34.5 protein is important for counteracting host antiviral responses to allow viral replication in the nervous system [9], [10]. It is required for complete virulence in the adult mouse brain [9], [10], and alters host responses through the type I interferon (IFN), PKR, and RNAse L signaling pathways during early infection [8]. Within the γ34.5 protein are domains that specifically target host translational arrest [11], [12] and type I IFN response induction through TANK-binding kinase 1 (TBK1) [13], [14]. Recently, γ34.5 has also been shown to specifically inhibit initiation of autophagy in infected cells [15], [16].

Autophagy is critical for control of neurotropic viruses, including HSV, in the murine CNS [16]–[19]. This mechanism contributes to innate antiviral responses, and is thought to be particularly important in post-mitotic cells such as neurons to avoid cell death. Sensing of viral nucleic acid in an infected cell initiates type I IFN responses, activating the double-stranded RNA (dsRNA)-dependent protein kinase PKR which in turn induces autophagy [15]. The HSV γ34.5 protein binds and inhibits the autophagy initiating protein beclin 1, counteracting the host autophagic response [16], [19]. Production of γ34.5 in cells potently inhibits autophagy, preventing formation of the characteristic LC3-GFP puncta in serum-starved cells [17].

Autophagy is important in normal neonatal physiology, and rapid upregulation of the autophagic machinery shortly after birth is required for survival in response to the sudden interruption in nutrient supply [20]. Proper regulation of autophagy is required for normal brain development in the neonate, with functional deficiencies in beclin 1 regulatory proteins leading to poor control of proliferation of CNS cells and excessive apoptotic cell death [21]. Although the basal levels of autophagy are elevated in the critical neonatal period of neurodevelopment, little is known about the neonatal autophagic response in the context of infection.

We demonstrate here that in contrast to the adult, the type I IFN response does not alter the outcome of HSV infection in the neonatal mouse brain. However, the HSV γ34.5 protein involved in countering type I IFN responses is required for full virulence in the neonatal mouse brain. Further investigation of a specific function of γ34.5 revealed that the autophagy inhibiting function of this protein, while important for neuropathogenesis in the adult, is dispensable for disease in the neonatal murine brain. Unlike in the adult, autophagy is activated in the neonatal brain during HSV infection and this activation is independent of type I interferon signaling. Additionally, we provide evidence that autophagy may be activated in human neonatal HSV encephalitis. Our findings suggest development-specific differences in the induction and regulation of autophagy during HSV infection of the CNS.

## Results

### Outcomes of HSV-1 infection in the neonatal mouse brain are not altered by type I IFN signaling

**Figure 1:**
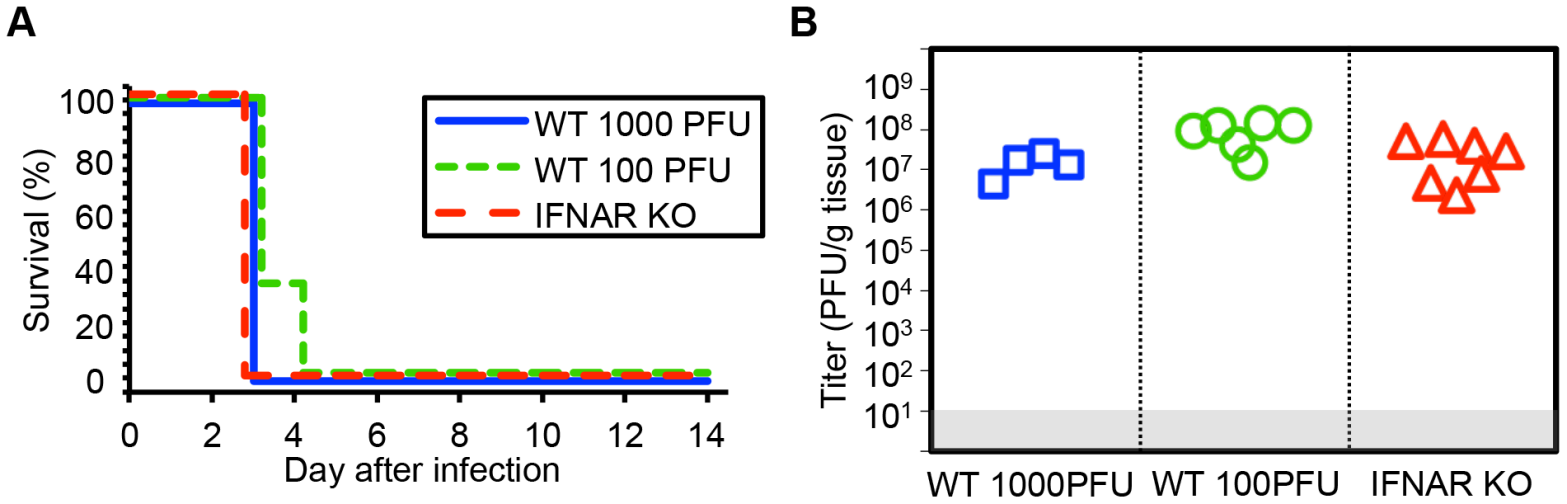
Type I interferon signaling does not alter mortality or control viral replication in the neonatal mouse brain after HSV infection. (A) Survival of 7-day-old (neonatal) WT mice inoculated with 10^3^ PFU HSV-1 F (n = 5), neonatal IFNAR KO mice (n = 9) inoculated with 10^3^ PFU of HSV-1 F, or neonatal WT mice inoculated with 100 PFU HSV-1 F (n = 6). Results shown represent survival data combined from 6 independent inoculations. Log-rank analysis showed no difference in time to mortality between groups of mice inoculated with 10^3^ PFU HSV-1 (*P* = 1.00) or between groups of WT mice inoculated with different amounts of virus (*P* = 0.17, median survival = 3 days in all groups). (B) HSV-1 titer in brain tissue of neonatal WT (10^3^ or 100 PFU) or IFNAR KO (10^3^ PFU) mice at death or post-inoculation day 14. Log-transformed mean titer at death did not differ between IFNAR KO and WT mice inoculated with 10^3^ PFU of HSV-1 (10^7.2^ PFU/g vs 10^7.1^ PFU/g, respectively, *P* = 0.67, *t* test). Log-transformed mean titer at death in brain homogenates from WT neonates inoculated with 100 PFU were statistically higher than those inoculated with 10^3^ PFU (10^7.3^ PFU/g vs 10^7.1^ PFU/g, respectively, *P* = 0.01).

Prior data from our group and others suggests that host immune responses may contribute to pathology after HSV infection in the newborn CNS [22]–[24]. We used viruses lacking specific genes, which interact with the type I inflammatory response, and mice lacking specific inflammatory signals, to test whether newborn disease would be altered after HSV infection. To determine the contribution of the type I IFN response to the pathogenesis of HSV in the neonatal brain, we inoculated 7-day-old wild-type (WT) or type I IFN receptor knockout (IFNAR KO) mice intracranially (IC) with HSV-1. Both WT and IFNAR KO newborns had 100% mortality by 3 days after inoculation with 1000 PFU virus (Fig. 1A). Viral titer in the neonatal brain at mortality was equivalent independent of intact type I IFN signaling (Fig. 1B). To confirm this was not an inoculum effect, we inoculated WT neonatal mice at a ten-fold lower dose of 100 plaque-forming units (PFU). There were no differences in time to mortality or overall mortality in the CNS after inoculation with 100 PFU compared to 10^3^ PFU (Fig. 1A, 1B), further illustrating the exceptional susceptibility of the neonatal brain to infection. Mean log-transformed viral titer was between 10^7^ and 10^9^ PFU/g in the groups receiving different inocula, and was actually statistically higher in the group, which received less virus.

### The γ34.5 protein of HSV-1 F is required for full virulence after IC inoculation of newborn and adult mice

**Figure 2:**
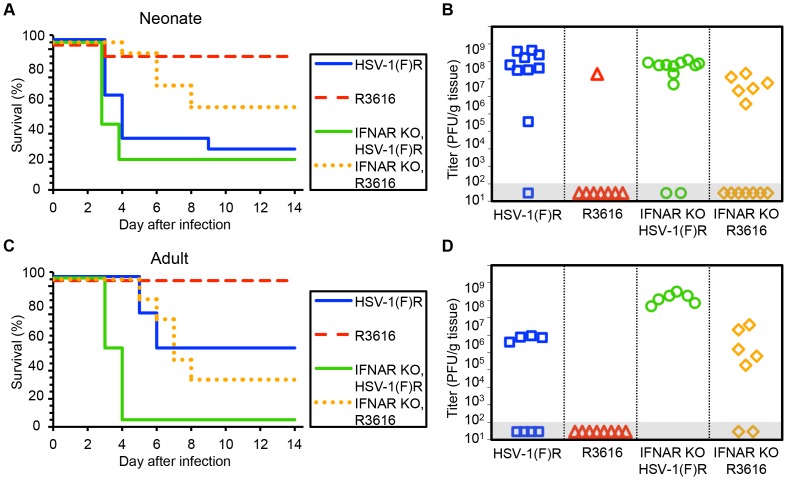
The HSV-1 F γ34.5 protein is important for mortality and viral replication in the CNS of both neonatal and adult mice. (A) Survival of 7-day-old WT (n = 11–13) or IFNAR KO (n = 12–13) littermates after IC inoculation with 10^3^ PFU of either the γ34.5-null HSV-1 F-strain mutant (R3616) or its marker rescue (HSV-1(F)R). Results shown represent survival data combined from 9 independent inoculations. Log-rank analysis showed a statistically significant delay in time to mortality for WT newborns (*P* = 0.0015), or for IFNAR KO newborns (*P* = 0.003), after inoculation with R3616 as compared with HSV-1(F)R (median survival  = 4 days (WT) and 3 days (IFNAR KO)). (B) Titer of R3616 or HSV-1(F)R in the brain tissue of neonatal WT or IFNAR KO mice at death or post-inoculation day 14. Titers at death for R3616 in IFNAR KO neonates were significantly lower than for HSV-1(F)R (10^6.6^ PFU/g vs 10^7.7^ PFU/g, respectively, *P* = 0.005). (C) Survival of adult WT (n = 8 in each group) or IFNAR KO (n = 6–7) mice inoculated IC with 10^4^ PFU of either R3616 or HSV-1(F)R. Results shown represent survival data combined from 7 independent inoculations. Log-rank analysis showed a statistically significant delay in time to mortality for WT adults (*P* = 0.025) or IFNAR KO adults (*P* = 0.0004); after inoculation with R3616 as compared with HSV-1(F)R (median survival  = 10 days (WT) and 3.5 days (IFNAR KO)). (D) Titer of R3616 or HSV-1(F)R in the brain tissue of adult WT or IFNAR KO mice at death or post-inoculation day 14. R3616 was not detected at day 14 after infection in brain homogenates from WT adult mice, and titers at death for R3616 in IFNAR KO adults were significantly lower than for HSV-1(F)R (10^5.4^ PFU/g vs 10^8.1^ PFU/g, respectively, *P* = 0.003).

Our finding that the type I IFN response did not protect newborns from HSV disease suggested that a viral factor important to counteracting this response might be dispensable for CNS pathogenesis in this population. We inoculated 7-day-old newborn mice with an HSV-1 F strain mutant deleted in both copies of the γ34.5 gene (R3616) or its marker rescue (HSV-1(F)R) [9]. Unexpectedly, the R3616 virus was significantly attenuated for mortality in the WT neonate (Fig. 2A). Replication was also defective in the CNS of WT newborns for the R3616 virus, which was detected from the brain of only one pup (Fig. 2B). Interestingly, several WT pups infected with R3616 had weight loss at day 9–10 after inoculation as the only detectable clinical symptom, with all affected pups regaining lost weight by day 14. Removing type I IFN signaling in the newborn mouse restored some virulence to the R3616 virus, but it remained attenuated for both mortality and replication relative to HSV-1(F)R (Fig. 2A, 2B). For both WT and IFNAR KO newborns, mortality after IC inoculation and replication of the marker rescue virus HSV-1(F)R in the CNS were comparable to WT HSV-1 F.

Results in adult mice inoculated IC with R3616 or HSV-1(F)R were consistent with prior studies [9] and comparable to our observations in newborn mice, with no mortality or CNS replication in WT adults after inoculation with R3616 (Fig. 2C, 2D). Mortality and replication of R3616 in adult IFNAR KO mice demonstrated a similar phenotype as observed in newborns, with some restoration of virulence and viral replication, though not to the same extent as for inoculations with HSV-1(F)R. In contrast to our observations in newborns, adult mice demonstrated a significant dependence on type I IFN signaling after IC inoculation with WT virus, as has been previously shown [25]. WT adult mice had a 50% survival rate after inoculation with 10^4^ PFU HSV-1(F)R, with a median time to death of 10 days (Fig. 2C), while adult IFNAR KO mice had 100% mortality and a median survival time of 3.5 days. Among mice that died, HSV-1 titer in the brain was more than 100 fold higher in IFNAR KO adult mice inoculated with HSV-1(F)R compared to WT adult mice (Fig. 2D). These data suggest that the type I IFN response makes a larger contribution to control of WT HSV infection in the adult mouse brain than in the newborn brain, but that HSV-1 F γ34.5 is important for neurovirulence in both age groups. Genetic ablation of type I IFN signaling did not restore full virulence to the R3616 mutant virus in either age group.

### The γ34.5 protein of HSV-1 strain 17 is required for full virulence after IC inoculation of WT newborn and adult mice

**Figure 3:**
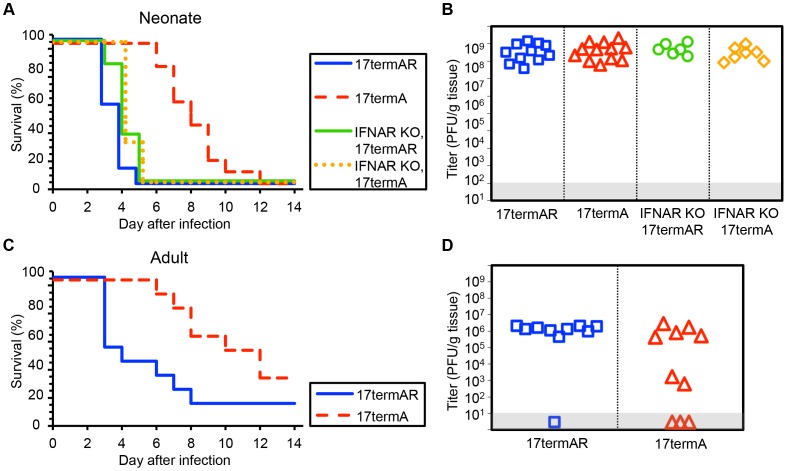
HSV-1 strain 17 γ34.5 is important for mortality and viral replication in the CNS of WT neonatal and adult mice, but virulence is restored in neonates with disrupted type I IFN signaling. (A) Survival of 7-day-old WT (n = 11–12) or IFNAR KO (n = 6–7) littermates after IC inoculation with 10^3^ PFU of either the γ34.5-null HSV-1 strain 17 mutant (17termA) or its marker rescue (17termAR). Results shown represent survival data combined from 7 independent inoculations. Log-rank analysis showed a statistically significant delay in time to mortality for WT newborns (*P*<0.0001, median survival  = 8 days), but not for IFNAR KO newborns (*P* = 0.83, median survival  = 4 days), after inoculation with 17termA as compared with 17termAR. There was no difference in time to mortality between WT and IFNAR KO neonatal mice inoculated with 17termAR (*P* = 0.16, median survival  = 4 days in both groups). (B) Titer of 17TermA or 17TermAR in the brain tissue of neonatal WT or IFNAR KO mice at death. Log-transformed mean titers did not differ in pairwise comparisons between any groups (10^8.5^ PFU/g for WT+17termAR, 10^8.6^ PFU/g for WT+17termA, 10^8.7^ PFU/g for IFNAR KO+17termAR, 10^8.4^ PFU/g for WT+17termA, *P*>0.2). (C) Survival of adult WT mice inoculated IC with 10^4^ PFU of either 17termA or 17termAR (n = 10 in each group). Results shown represent survival data combined from 2 independent inoculations. Log-rank analysis showed a statistically significant delay in time to mortality after inoculation with 17TermA (median survival  = 11 days) as compared with 17termAR (*P* = 0.01, median survival  = 3.5 days). (D) Titer of 17termA or 17termAR in the brain tissue of adult WT mice at death or post-inoculation day 14 (10^5.1^ PFU/g vs 10^6.1^ PFU/g, respectively, *P* = 0.13).

HSV-1 F is a virulent strain of HSV initially isolated from a facial lesion [26]. As the phenotypic characteristics of a mutant virus may depend on the genetic background in which the mutation is made [10], we conducted similar experiments using viruses on the HSV-1 strain 17 genetic background. WT newborns inoculated with an HSV-1 strain 17 mutant 17termA, which lacks both copies of γ34.5, had delayed mortality relative to littermates inoculated with the marker rescue virus 17termAR (Fig. 3A). However, unlike our observations with viruses on the F background, these mice eventually succumbed to infection, and both viruses were present at similar levels in brain homogenates at the time of death (Fig. 3B). Interestingly, unlike our observation using R3616, genetic deletion of the type I IFN receptor completely restored virulence of 17termA at the inoculum tested, with comparable replication of both viruses in the CNS of IFNAR KO pups (Fig. 3A, 3B). Results in WT adult mice inoculated IC with 17termA or 17termAR were consistent with prior studies [10] and comparable to our observations in newborn mice, with delayed mortality and evidence of CNS replication in WT adults after inoculation with 17termA (Fig. 3C, 3D). Together, these data indicate that the HSV γ34.5 protein plays a critical role in the pathogenesis of WT HSV in both the neonatal and adult brain in vivo. However, the restoration of virulence of the 17termA mutant in IFNAR KO newborns suggests that the type I IFN signaling pathway is induced during infection in the newborn brain, though not to an extent which controls disease from WT virus.

### Inhibition of autophagy through beclin 1 is dispensable for HSV pathogenesis in the neonatal CNS

**Figure 4:**
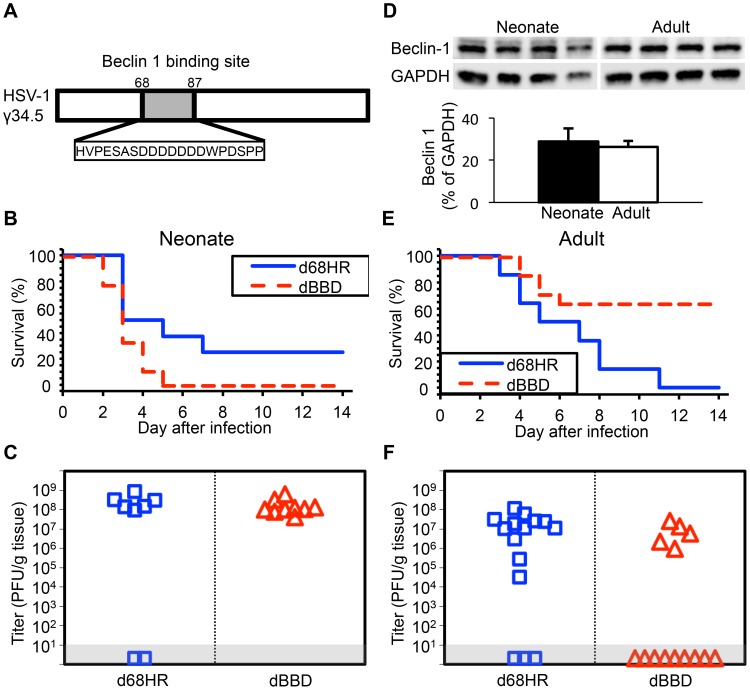
Inhibition of autophagy through beclin 1 binding is dispensable for HSV-1 pathogenesis in the neonatal mouse brain. (A) Diagram of the HSV-1 γ34.5 protein indicating domain deleted to create the dBBD virus. (B) Survival of 7-day-old WT littermates inoculated IC with 10^3^ PFU of either the HSV-1 mutant deleted in the 20 amino acid sequence of γ34.5 required for beclin 1 binding (dBBD, n = 9), or its marker rescue (d68HR, n = 8). Results shown represent survival data combined from 5 independent inoculations. Log-rank analysis showed a statistically significant delay in time to mortality after inoculation with d68HR (median survival  = 4 days) compared to dBBD (*P* = 0.04, median survival  = 3 days). (C) Titer of dBBD or d68HR in the brain tissue of neonatal WT mice at death or post-inoculation day 14. Of those that died from HSV disease, no differences in log-transformed mean titer were identified between the two groups (10^8.1^ PFU/g for dBBD and 10^8.4^ PFU/g for d68HR, *P* = 0.19). (D) Representative beclin 1 immunoblots (top) and densitometry (bottom, mean values ± standard deviation) of whole-brain homogenates from uninfected 7-day-old neonatal and 10-week-old adult mice (n = 4 in each group). (E) Survival of adult WT mice inoculated IC with 10^4^ PFU of either dBBD (n = 14) or d68HR (n = 14). Results shown represent survival data combined from 5 independent inoculations. Log-rank analysis showed a statistically significant delay in time to mortality after inoculation with dBBD (median survival  =  undefined) compared to d68HR (*P* = 0.005, median survival  = 6 days). (F) Titer of dBBD or d68HR in the brain tissue of adult WT mice at death or post-inoculation day 14. No differences in log-transformed mean titer between the two groups were identified among animals that that died from HSV disease (10^6.7^ PFU/g for dBBD and 10^6.9^ PFU/g for d68HR, *P* = 0.64).

Our data suggest limited contribution of type I IFN signaling to protection of newborns against CNS disease from WT HSV-1, but involvement of HSV-1 γ34.5 in promoting CNS disease in both newborns and adults. Type I IFN signaling activates PKR, which promotes induction of autophagy [15]. HSV γ34.5 directly inhibits autophagy via an interaction with beclin 1, contributing to CNS disease in adult mice [16]. A possible explanation for our observations in the newborn is that an already blunted type I IFN response is completely suppressed by WT HSV-1 in the CNS, and autophagy is not sufficiently activated to provide protection against CNS disease. Deletion of γ34.5 could allow enough type I IFN signaling to promote activation of autophagy, which is not suppressed by the virus, attenuating disease. To specifically determine the importance of HSV modulation of the autophagic pathway through beclin 1 to pathogenesis in the neonatal CNS, we inoculated mice using a mutant virus (dBBD) containing a deletion in γ34.5 of 20 amino acids required for beclin 1 binding (Fig. 4A), compared with its rescue virus d68HR. Interestingly, neonatal mice inoculated with the dBBD mutant virus had a slightly shorter time to mortality and an increased overall mortality compared to d68HR (Fig. 4B). Furthermore, viral replication of the dBBD virus in the neonatal mouse brain was similar to the d68HR rescue virus (Fig. 4C). These observations were not due to absence of production of beclin 1 in newborn brains, as protein was detected at similar levels to adult mice (Fig. 4D). In contrast to newborns, adult mice inoculated with the dBBD virus had a significantly increased overall survival compared with adult mice inoculated with d68HR (Fig. 4E), and replication of dBBD in the adult CNS was reduced relative to d68HR (Fig. 4F), consistent with prior reports [16]. These results demonstrate that a domain of HSV γ34.5 important for binding beclin 1 to inhibit autophagy is dispensable for mortality and viral replication in the neonatal mouse brain, but important in the adult.

### Autophagy is activated in the neonatal mouse brain during HSV infection

**Figure 5:**
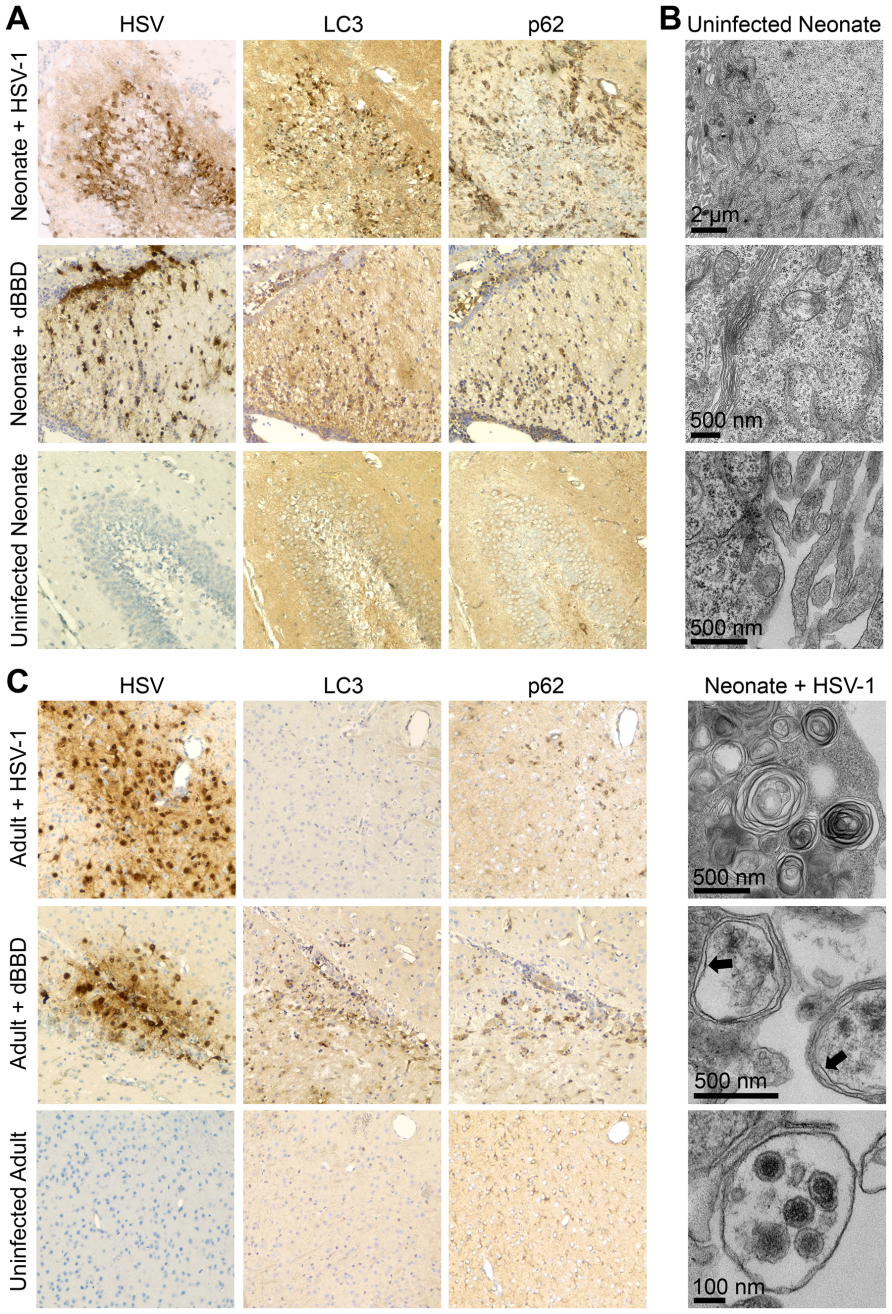
Autophagy in the CNS is activated in the neonatal mouse during HSV infection, but is effectively inhibited in the adult. (A) Representative immunohistochemical analysis (original magnification: 200x) of neonatal WT mice inoculated IC with 10^3^ PFU of either WT HSV-1 F (top row) or mutant HSV-1 deleted for beclin 1 binding (dBBD, middle row). Mice were followed until neurologic symptoms developed on day 3. Paraffin-embedded brains were serially sectioned and stained for HSV-1 or markers of activated autophagy (LC3 and p62). A representative image of an HSV-infected region in the neonate (top) is positive for markers of autophagy. The dBBD-infected neonatal brain also stained positive for LC3 and p62 in serial slides from infected regions (middle row). The uninfected neonatal brain is negative for all markers (bottom). (B) Representative electron micrographs of neonatal brains. In HSV-infected cells, double-membrane autophagosomes are abundantly present (bottom panel, indicated by arrows), and HSV intact and degraded virions are found in autophagolysosomes (bottom). These structures are absent in mock-infected brain (top). (C) Representative immunohistochemical analysis (original magnification: x400) of adult WT mice inoculated IC with 10^4^ PFU of either WT HSV-1 F (top row) or dBBD (middle row). Mice were followed until neurologic symptoms developed on day 4. A representative image of an HSV-infected region (top) is negative for markers of autophagy. The dBBD-infected areas colocalized with positive markers of autophagy on serial sections (middle row). Control uninfected adult mice were negative for all markers (bottom).

The observation that a beclin binding domain of HSV-1 γ34.5 is dispensable for HSV pathogenesis in newborn brains (Fig. 4) is consistent with the hypothesis that there is defective activation of autophagy by the newborn host after infection. To investigate the induction of autophagy in the newborn CNS after infection, we inoculated WT neonatal mice IC with WT HSV-1 and sacrificed them for immunohistochemical (IHC) analysis three days later, at the height of neurologic symptoms but prior to mortality. Several regions in the neonatal CNS stained positive for HSV antigen, which was most commonly detected in the hippocampus, caudate, putamen, and cerebellum (Fig. 5A). Sections adjacent to those found positive for HSV were evaluated further for the autophagy markers LC3 and p62. Surprisingly, these markers were floridly positive in the neonatal mouse brain after infection with WT virus, which retains beclin 1 binding activity. Abundant LC3 and p62 were similarly detected in infected regions of neonatal mice infected with the dBBD virus (Fig. 5A, middle panel), as would be expected with a virus unable to inhibit autophagy. Similar staining of brain regions from control uninfected neonatal mice lacked immunoreactivity for LC3 and p62. IHC analyses of similarly infected adult brains demonstrated HSV antigen in several different brain regions, including the hippocampus, caudate, putamen, cerebellum, periaqueductal gray, and cortex. However, in adjacent sections these regions were absent for p62 and LC3 when WT virus was used (Fig. 5C, top row), but detectable in adult brains after infection with mutant HSV-1 deleted for beclin 1 binding (dBBD) (Fig. 5C, middle row), consistent with the expected inhibition of autophagy by WT but not dBBD virus.

Although LC3 is specifically incorporated into the developing autophagosome [27],[28], detection by IHC analysis indicates activation of the autophagic process, but not completion. To demonstrate formation of mature autophagosomes in the neonatal brain during HSV infection [29], we imaged infected tissue by transmission electron microscopy. We detected abundant cytoplasmic double membrane vesicles characteristic of autophagosomes, containing electron-dense bodies consistent with HSV virions (Fig. 5B). Taken together, these results demonstrate autophagy is activated in the murine neonatal brain after infection with either WT or dBBD HSV-1, but in adult brains only when HSV-1 is unable to interact with beclin 1.

### Autophagy is activated during HSV infection independent of the type I interferon response in the newborn, but not adult, mouse brain

**Figure 6:**
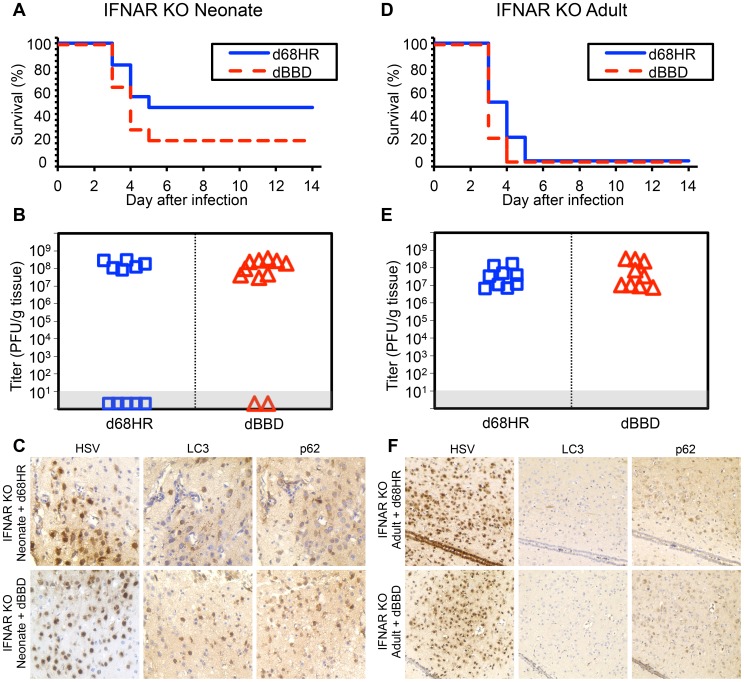
Autophagy is activated during HSV infection independent of type I interferon signaling in the newborn, but not the adult, murine CNS. (A) Survival of 7-day-old IFNAR KO littermates inoculated IC with 10^3^ PFU of either the dBBD mutant virus or its marker rescue d68HR (n = 11 in each group). Results shown represent survival data combined from 4 independent inoculations. Log-rank analysis showed no statistically significant difference in time to mortality between d68HR and dBBD-inoculated IFNAR KO neonatal mice (*P* = 0.25, median survival  = 4 days in both groups) (B) Titer of dBBD or d68HR in the brain tissue of neonatal IFNAR KO mice at death or post-inoculation day 14. Of the mice that died from HSV disease, no differences in log-transformed mean titer were identified between dBBD and d68HR-infected mice (10^8.1^ PFU/g vs 10^8.2^ PFU/g, respectively, *P* = 0.5). (C) Representative immunohistochemical analysis (original magnification: x400) of neonatal IFNAR KO mice inoculated IC with 10^3^ PFU of either d68HR (top row) or dBBD (bottom row). Mice were followed until neurologic symptoms developed on day 3. The representative images of d68HR and dBBD-infected regions are positive for markers of autophagy on serial sections. (D) Survival of adult IFNAR KO mice inoculated IC with 10^4^ PFU of either dBBD or d68HR (n = 10 in each group). Results shown represent survival data combined from 6 independent inoculations. Log-rank analysis showed no statistically significant difference in time to mortality after inoculation with dBBD (*P* = 0.1, median survival  = 3 days in both groups). (E) Titer of dBBD or d68HR in the brain tissue of adult IFNAR KO mice at death or post-inoculation day 14. Log-transformed mean titer was not significantly different between adult IFNAR KO mice infected with dBBD or d68HR (10^7.6^ PFU/g vs 10^7.4^ PFU/g, respectively, *P* = 0.5). (F) Representative immunohistochemical analysis (original magnification: x200) of adult IFNAR KO mice inoculated IC with 10^4^ PFU of either d68HR (top row) or dBBD (bottom row). Mice were followed until neurologic symptoms developed on day 3. The representative images of d68HR and dBBD-infected regions are negative for markers of autophagy on serial sections.

We have presented evidence that autophagy is activated in the neonatal brain during HSV infection (Fig. 5), despite data supporting a limited contribution of type I IFN signaling to protection of newborns against CNS disease from WT HSV-1 (Fig. 1–3). This led us to investigate the contribution of type I IFN signaling to activation of autophagy in the newborn brain. We inoculated IFNAR KO neonatal mice IC with either the dBBD mutant virus or its marker rescue, d68HR. Similar to our findings in WT neonatal mice (Fig. 4), there was no difference in time to mortality or overall mortality between dBBD and d68HR in IFNAR KO neonatal mice (Fig. 6A). Furthermore, viral titers in the IFNAR KO brain were similar at mortality in mice inoculated with the beclin binding mutant HSV or its marker rescue (Fig. 6B). Since autophagy is activated in the WT newborn CNS despite an apparently blunted type I IFN response, we investigated the activation of autophagy in IFNAR KO newborns during HSV infection. Both LC3 and p62 were abundantly positive by IHC on serial sections in IFNAR KO neonatal brains infected with either dBBD (Fig. 6C, bottom panel) or d68HR (Fig. 6C, top panel). These data suggest that intact type I IFN signaling is not required for activation of autophagy in the neonatal murine brain during HSV-1 infection. In contrast, in the adult the induction of PKR activity by type I IFN signaling provides a link between type I IFN and activation of autophagy [29]. To confirm this link in the adult brain, we inoculated adult IFNAR KO mice IC with either dBBD or d68HR. HSV-1 suppression of autophagy is not required for virulence in IFNAR KO adults, as mortality from the dBBD mutant was equivalent to the rescue virus levels (Fig. 6D). Additionally, viral titers at mortality were similar for the two viruses in these experiments (Fig. 6E). Consistent with the expectation that type I IFN signaling is required for induction of autophagy, infected IFNAR KO adult mouse brains were absent by immunohistochemical staining for positive markers of autophagy after inoculation with either dBBD or d68HR (Fig. 6F). Taken together, these results suggest that type I IFN signaling is dispensable for effective activation of autophagy in the neonatal mouse brain after HSV-1 infection, but required in the adult mouse brain.

### Activation of autophagy in the HSV-infected newborn brain is associated with markers of apoptosis

**Figure 7:**
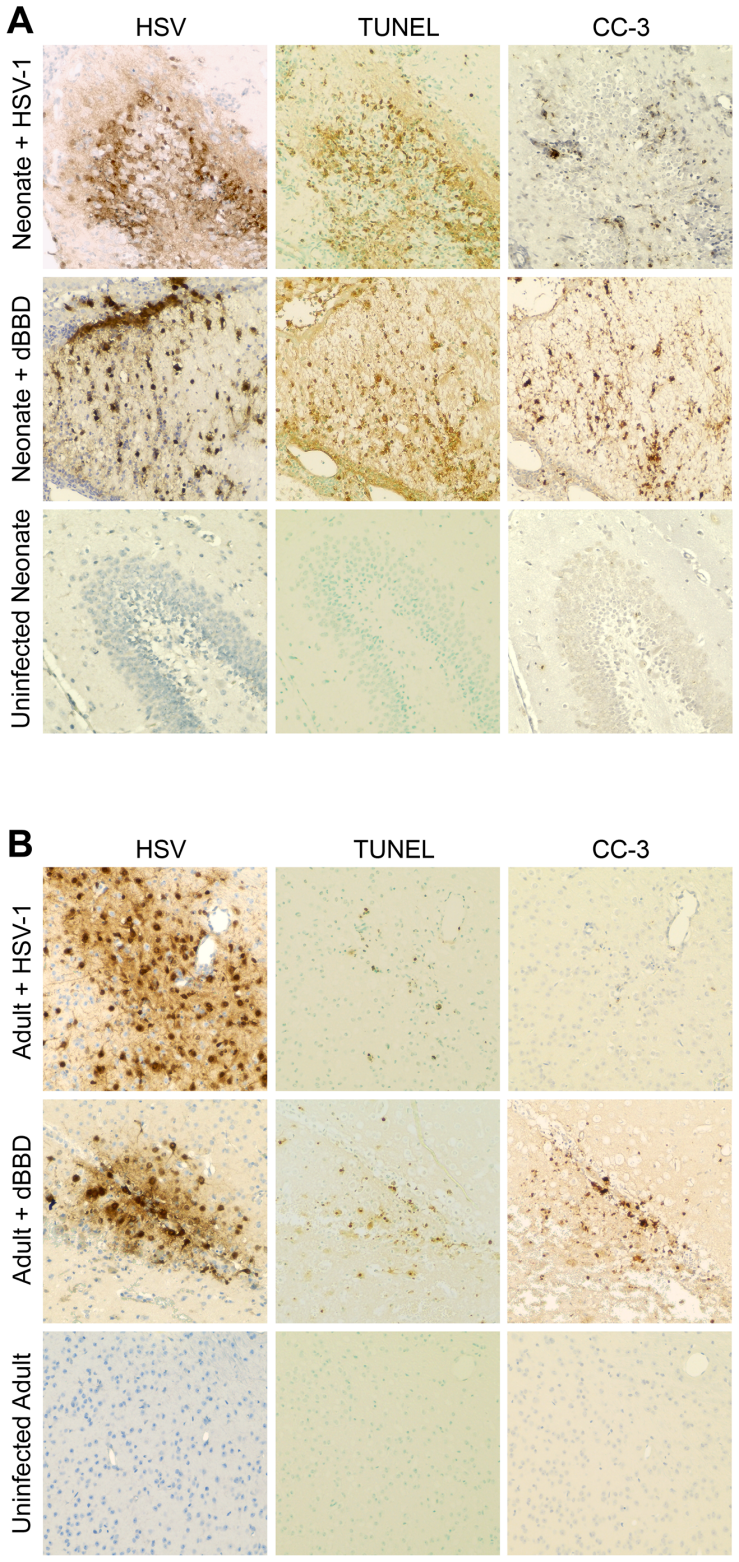
Infected regions with activated autophagy in the newborn mouse brain are associated with markers of apoptotic cell death. (A) Representative immunohistochemical analysis of neonatal murine brain infected with WT HSV-1 F (original magnification: 200x). The HSV antiserum panel from Fig. 5 is repeated for orientation. The WT HSV-infected neonatal brain is positive for TUNEL staining and caspase-3 activation (CC-3) at the site of infection (top). Representative sections from a neonatal murine brain infected with the dBBD virus also demonstrate positive TUNEL staining (middle) and cleaved caspase-3 (middle, right) in infected regions of the brain. The control uninfected neonatal brain is negative for markers of apoptosis (bottom). (B) Serial sections of brain from an adult mouse infected with HSV-1 F demonstrate scant TUNEL staining and few detectable cleaved caspase-3 positive cells (top). The dBBD-infected adult murine brain is also scantly positive for TUNEL staining (middle), and cleaved caspase-3 was positive in serial sections. Control uninfected adult mouse brain sections are shown below (original magnification: 200x).

Studies of neonatal CNS diseases such as hypoxic-ischemic injury suggest an association between activation of autophagy and apoptotic cell death in the brain [30]. To investigate whether there was similar evidence of apoptosis in regions of the infected newborn brain which demonstrated activated autophagy, we performed TUNEL staining in serial sections from infected mouse brain samples. TUNEL staining was abundant in areas of the neonatal mouse brain infected with wild-type HSV-1 or dBBD (Fig. 7A), but only scantly positive in a region heavily infected with HSV-1 or dBBD in the adult murine brain (Fig. 7B). To further confirm that the DNA fragmentation observed in the infected neonatal brain is due to apoptosis, we stained additional sections for cleaved caspase-3, a specific marker of apoptotic cell death [31]. Cleaved caspase-3 was detected in regions of the brain in which TUNEL-positive staining was observed (Fig. 7A). Moreover, cells immunoreactive for cleaved caspase-3 were more abundant in the infected neonatal brain than in the adult brain (Fig. 7B). Morphologically, the caspase-3 positive cells were consistent with neurons and adjacent glial cells. Interestingly, foci of apoptotic cells in the brains of HSV-infected neonatal mice coincided with areas of intense staining for autophagic markers, suggesting a link between activation of autophagy and cellular apoptosis during HSV infection of the developing brain.

### Autophagy and apoptosis are activated in a case of human neonatal HSV encephalitis

**Figure 8:**
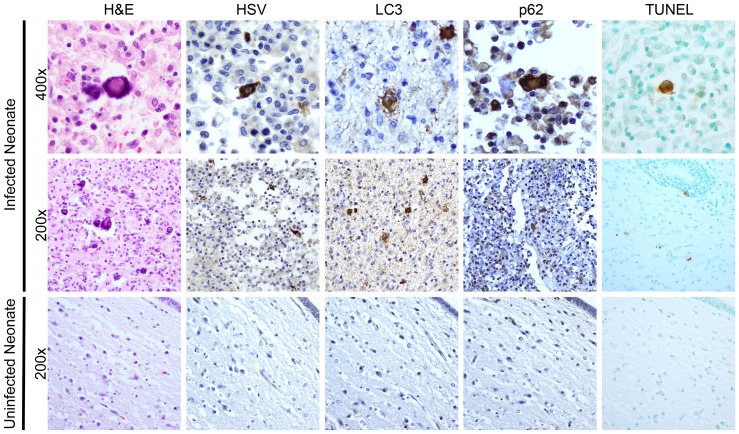
Activation of autophagy and apoptotic cell death in a human case of neonatal HSV encephalitis. H&E staining of the thalamus in case of neonatal HSV encephalitis demonstrates cytopathic effect (CPE) pathognomonic of HSV infection (top, middle left). In serial sections, these same cells were positive for HSV-antigen. Markers of activated autophagy (LC3 and p62) were present in multinucleated cells with CPE characteristic of HSV infection (top row). Infected cells were positive for TUNEL staining (far right). Control thalamic tissue from a neonate without HSV infection was negative for HSV, LC3, and p62 and demonstrated only scantly positive TUNEL staining (bottom row).

Although animal models of disease often enhance our general understanding of disease pathogenesis in mammalian hosts, we sought to confirm our observations of activated autophagy and apoptosis in newborn HSV encephalitis by studying sections of brain from a human case of neonatal HSV encephalitis. Staining of infected human brain tissue with hematoxylin and eosin (H&E) revealed several cells exhibiting cytopathic effects consistent with HSV infection, including eosinophilic nuclear inclusions, nuclear swelling, and multinucleate cells (Fig. 8, top row). Cells displaying these morphologic changes consistent with HSV infection stained positive for HSV antigen. These HSV antigen-positive cells were morphologically consistent with neuronal and microglial cells. Furthermore, infected cells in adjacent sections were positive for the markers of activated autophagy LC3 and p62 (Fig. 8, top row), which displayed the granular cytosolic appearance characteristic of autophagic punctae. Regions of activated autophagy also demonstrated TUNEL-positive staining. In comparison, analysis of a similar region of brain tissue at autopsy from a human neonate which died of non-neurologic disease was negative for cytopathic effect by H&E, and staining was negative for markers of autophagy or for HSV antigen (Fig. 8, bottom row). TUNEL staining in the control sample was only scantly positive. Collectively, these results demonstrate that autophagy and cellular apoptosis are activated during human neonatal HSV encephalitis.

## Discussion

Here we report an age-dependent difference in autophagy as a mechanism for protection from HSV encephalitis. Our key findings are: (a) in the newborn brain, HSV remains virulent independent of its ability to bind and inhibit beclin 1 (Fig. 4); (b) CNS disease from HSV occurs in newborn brain despite the activation of autophagy, which is not inhibited by virus possessing the ability to bind beclin 1 (Fig. 5); and (c) type I IFN signaling is not required to initiate autophagy in the newborn brain after infection (Fig. 6). These observations stand in sharp contrast to the situation in the adult brain, in which autophagy is stimulated by type I IFN responses, and HSV-1 requires suppression of autophagy to promote disease.

Inflammatory signaling via type I IFN in newborns is generally blunted compared with adults [32], and consistent with this observation, we found that signals mediated by the type I IFN receptor in the neonate did not alter the outcome of WT HSV-1 infection in the CNS (Fig. 1–3). However, deletion of the HSV-1 protein γ34.5, which interacts at different points with PKR-dependent signals and participates in countering host type I IFN responses [12], [33]–[36], attenuated CNS disease in both neonatal and adult WT mice (Fig. 2–3). Additionally, IFNAR KO newborns were equally susceptible to disease from virus deleted of γ34.5 on the strain 17 background as to the WT virus (Fig. 3), suggesting that type I IFN responses are not completely absent in the newborn CNS, but are likely to be more easily overcome by suppressive mechanisms present in HSV-1 than are similar signals in the adult CNS. The γ34.5 protein is multifunctional and includes domains that interact with different host signaling pathways, including signals mediated by TANK-binding kinase-1 [13], [32], the protein phosphatase PP1α to counter host translational arrest mediated by PKR [11], [12], and beclin 1 for inhibition of autophagy [15], [16]. Although we have demonstrated that the autophagy inhibiting function of γ34.5 is dispensable for pathogenesis of HSV in the neonatal CNS, the importance of γ34.5 in mediating disease suggests that the other functions of this protein contribute to disease in the developing brain, an active area of investigation.

Activation of autophagy during inflammatory responses typically involves PKR-dependent mechanisms that are augmented by type I IFN signaling [37], [38]. We observed this in experiments in adult mice, where absence of the type I IFN receptor resulted in absence of LC3 staining in the CNS (Fig. 6F) as compared with WT adult mice (Fig. 5C). In distinct contrast, HSV infection of the neonatal brain activated autophagy independent of type I interferon signaling (Fig. 6C). Moreover, deletion of a domain of γ34.5 responsible for binding beclin 1 did not suppress autophagy in the newborn brain (Fig. 5), consistent with the hypothesis that a beclin 1-independent mechanism promotes autophagy initiation during inflammatory responses in the neonatal brain. Although beclin 1 is considered to be the central initiating protein for autophagy, in some circumstances it may be dispensable for activation of autophagy [39], [40]. Recent studies suggest that the cellular prior protein (PrP) is a positive regulator of autophagy in the CNS during HSV infection [41]. Notably, this study demonstrated that genetic deletion of PrP in adult mice restored virulence of a beclin 1 binding mutant to wild-type HSV-1 levels. Furthermore, in vitro replication of the BBD mutant in PrP-knockout cells was only observed in glial cells, and not mature neurons, consistent with our previous observation that mature neurons are not the primary target of HSV infection in the neonatal CNS [23]. Finally, PrP is produced early in life in the rodent brain [42], and inflammatory signals outside of type I IFNs may induce PrP production in the brain [43]. Together, these observations suggest that the newborn brain may respond to infection with production of cytokines that promote PrP, which in turn may stimulate induction of autophagy. This provides a possible mechanism for type I IFN-independent, beclin 1-independent promotion of autophagy as we observed in the newborn brain.

We observed an association between activation of autophagy and increased apoptosis in infected regions of the newborn but not adult brain (Fig. 7). Cellular regulation of these processes is complex and overlapping, with upstream events in both processes often triggered by the same signals [44], [45]. Although autophagy can inhibit the induction of cell death pathways, including apoptosis and necrosis [46], an association between excessive autophagy and cell death has been proposed, but whether cell death occurs because of autophagy or despite autophagy has been debated [47]. Consistent with our observations in the HSV-infected neonatal brain, models of neonatal hypoxic-ischemic injury identified an association between the activation of autophagy and the presence of apoptotic markers in the hippocampus [30]; knock-out of the essential autophagy gene ATG7 in this model decreased apoptotic cell death. The developing brain differs from the mature adult brain in having both increased neurogenesis [48] and increased neuronal apoptosis [49], as circuits are sculpted to ultimately create the networks which process information in the mature animal. Control of these processes is complex and not well understood [50], with even less known about the influence of infection and inflammation on these processes. Our data suggest that infection may perturb developmental regulation of cell death in the nervous system, possibly triggering apoptosis in additional cells during infection.

The increased susceptibility to infection in newborns has been the subject of a great deal of study, with numerous differences identified in immune defense of newborns compared with adults [32], [51]. Our study has identified a previously unappreciated difference in the newborn response to CNS infection relative to the adult, which is associated with increased cell death in the brain. Moreover, we provide evidence that our observations in a mouse model of infection are relevant to human disease, as activation of autophagy was demonstrated in a human case of neonatal encephalitis (Fig. 8). Combined with prior observations in a different newborn CNS disease [30], our results suggest that excessive autophagy in the developing brain may more generally contribute to newborn pathology. This distinction from the cytoprotective role of autophagy in the adult has important therapeutic implications. Development of autophagy inducing drugs could provide clinical benefit in adult CNS infections [52], but differential outcomes associated with development could lead to detrimental responses in a younger population. Future therapeutics in the newborn will need to be catered to the unique physiology of the developing brain.

## Materials and Methods

### Viruses and cells

The HSV-1 F-strain virus (kindly provided by Bernard Roizman, University of Chicago, Chicago, Illinois, USA) is a low-passage clinical strain of HSV-1 originally obtained from a facial lesion and isolated in Hep-2 cells [26]. The mutant HSV-1 deleted of both copies of γ34.5, R3616, and the rescue virus with both copies of γ34.5 restored, HSV-1(F)R, were also provided by Bernard Roizman and are previously described [9]. The mutant virus deleted of both copies of γ34.5 on the HSV-1 strain 17 background (17TermA) and its marker rescue with both copies of γ34.5 restored (17TermAR) were provided by Richard Thompson [10]. The HSV-1 virus deleted in the beclin 1-binding domain of γ34.5 encoding amino acids 68–87 (termed here dBBD) and its marker rescue control d68HR (kindly provided by David Leib, Dartmouth University, Lebanon, NH, USA) were constructed by homologous recombination [16], [53]. The recombinant HSV-1 expressing GFP from the UL3/4 intragenic region [54] was constructed by homologous recombination (kindly provided by Yasushi Kawaguchi, Nagoya University Graduate School of Medicine, Nagoya, Japan).

Vero cells were cultured in Dulbecco's modification of Eagle's (DME) medium plus 10% fetal bovine serum (FBS) and 1% penicillin-streptomycin, and were used for the propagation and titering of virus. Plaque titrations were performed by standard methods.

### Murine HSV encephalitis model

The mouse strains used have been previously described, including the 129S2 (WT) and interferon-α/β receptor knock-out (IFNAR KO) mice [55] on the 129S2 genetic background. Mice were maintained in specific-pathogen-free conditions until transfer to a containment facility just prior to infection.

Breeding pairs were regularly monitored, with males separated from gravid females prior to delivery. Pups were inoculated at seven days of age, which from an immunologic perspective corresponds most closely to humans at birth [56]. Virus was diluted in PBS containing 1% inactivated calf serum and 0.1% glucose (PBS-GCS) to deliver a target intracranial (IC) inoculum of 1×10^3^ PFU/pup. Infections of 8-10 week old adult mice were included for comparison with newborn infections, with target inocula of 1×10^4^ PFU/mouse.

For IC inoculation of either adult or newborn mice, a positive displacement syringe with a 26-gauge needle and a needle guard was used to inoculate 5 µL total volume into the brain. The needle was placed in the approximate region of the hippocampus, equidistant between the lambda and bregma through the left parietal bone lateral to the sagittal suture. Experiments also included control mice injected IC in an identical manner using the same volume PBS-GCS.

Infected mice were monitored daily for signs of neurologic disease, including lethargy, seizure, automatisms, ataxia, and hunched posture. Mice displaying severe signs of illness were immediately sacrificed. Brains were harvested from infected and control mice. Mice used for immunohistochemical analysis were perfused as described below. Tissues for titering were weighed, homogenized in DMEM with 5% inactivated calf serum and 1% ciprofloxacin, and sonicated. Tissue homogenates were stored at −70°C until analysis. All statistical analyses were performed using Prism 5.01 (GraphPad Software). Kaplan-Meier survival statistical analysis was performed using the log-rank (Mantel-Cox) test. Comparisons of viral titers between different groups of mice was done by Student's t-test, using log-transformed values.

### Histology, immunohistochemistry, TUNEL staining, and immunoblots

Anesthetized mice were subjected to intracardiac perfusion with 4% paraformaldehyde in PBS. Whole brains were removed and post-fixed in 4% paraformaldehyde and subsequently embedded in paraffin. Four-µm-thick sections were mounted on glass slides. Antigen retrieval was performed manually using either a high pH Tris or citric-acid based solution (Vector Labs) at 95°C for 10 minutes. IHC staining was performed with anti-HSV antigen (Dako) diluted 1∶5000, anti-LC3 (Nanotools) diluted 1∶400, anti-cleaved caspase-3 (Cell Signaling) diluted 1∶500, or anti-p62 (Abnova) diluted 1∶2000 with the Vectastain Elite ABC kit (Vector Labs). HRP labeled secondary antibodies were visualized after treatment with the chromagen diaminobenzidine (DAB, Vector Labs). Finally, the slides were washed in tap water, counterstained in Gill's Hematoxylin, and imaged with the EVOS XL core cell imaging system. Western blots were performed on whole brain homogenates using a 1∶1000 dilution of anti-beclin 1 antibody (BD Biosciences) and anti- GAPDH (Abcam) as a loading control. Blots were visualized and densitometry analysis was performed using the LI-COR Odyssey system.

Paraffin-embedded sections were assayed for DNA fragmentation using the TUNEL technique (EMD Millipore) and counterstained with Methyl Green (Vector Labs). H&E staining was performed using Gill's Hematoxylin and Eosin Y solution.

### Transmission electron microscopy

Neonatal mice were inoculated as previously described with a recombinant HSV-1 expressing GFP [54] to allow for identification of infected regions. Mice were sacrificed at post-inoculation day 3, the brains were removed, and infected areas were dissected under a GFP scope (EVOS). Fresh tissue was post-fixed in 2% paraformaldehyde, 2.5% glutaraldehyde, and 0.1 M cacodylate. Samples were embedded in acrylic resin and thin sectioned by standard protocols and imaged with the FEI Tecnai Spirit G2 120 kV TEM.

### Human samples

Permission for the use of human postmortem tissue for this study was obtained from the Ann & Robert H. Lurie Children's Hospital of Chicago Privacy Board, in accordance with US Federal Regulations 45 CFR 46.160 and 164. Samples of brain tissue were obtained at autopsy from a seven week old male who presented at one month of age with symptoms of encephalitis, found to be HSV-2 positive in cerebrospinal fluid and subsequently brain tissue, who received treatment with acyclovir prior to death secondary to neurologic devastation. Control tissue was obtained at autopsy from a two day old male delivered at 30 weeks gestational age, who suffered acute gastrointestinal perforation one day prior to death from respiratory failure. There were no identified neurologic or infectious complications contributing to the death of this patient.

### Ethics statement

Animal care and use in this study were in accordance with institutional and NIH guidelines, as set forth in the "Guide for the Care and Use of Laboratory Animals" (National Academies Press, 2011). Northwestern also accepts as mandatory the PHS "Policy on Humane Care and Use of Laboratory Animals by Awardee Institutions" and NIH "Principles for the Utilization and Care of Vertebrate Animals Used in Testing, Research, and Training." All studies were approved by the Northwestern University Animal Care and Use Committee under the Animal Welfare Assurance Number A3283-01, Protocol 2013–2054.
